# Genetic diversity and drug sensitivity studies on *Eimeria tenella* field isolates from Hubei Province of China

**DOI:** 10.1186/s13071-017-2067-y

**Published:** 2017-03-09

**Authors:** Li Tan, Yalin Li, Xin Yang, Qiyun Ke, Weiqiang Lei, Mudassar Niaz Mughal, Rui Fang, Yanqin Zhou, Bang Shen, Junlong Zhao

**Affiliations:** State Key Laboratory of Agricultural Microbiology, College of Veterinary Medicine, Huazhong Agricultural University, Wuhan, 430070 Hubei People’s Republic of China

**Keywords:** *Eimeria tenella*, Genetic diversity, Maduramycin, Decoquinate, Diclazuril

## Abstract

**Background:**

Avian coccidiosis is an intracellular intestinal parasitic disease, caused by intracellular intestinal parasites from the genus *Eimeria*, among which *Eimeria tenella* is one of the most pathogenic species and causes great economic losses. Frequent applications of anticoccidial drugs have resulted in the development of drug-resistance in *E. tenella*. In the present study, we sought to determine the genetic diversity of *E. tenella* isolates prevalent in chicken farms in Hubei Province of China and examine their sensitivity to three anticoccidial drugs. The results provide useful information for the prevention and control of coccidiosis in this region.

**Methods:**

*Eimeria tenella* oocysts were isolated from faecal samples collected from different commercial broiler production farms in Hubei Province, China. After oocyst sporulation and animal inoculation for expansion of the field isolates, DNA and RNA were extracted from excysted sporozoites for molecular characterization. Species identification of field isolates were performed by polymerase chain reaction (PCR) amplification of the internal transcribed spacer 1 (ITS1) region of ribosomal DNA. Random amplified polymorphic DNA (RAPD) was used for population genetic analysis. Subsequently, sequences of the major sporozoite surface antigen (SAG), micronemal protein 2 (MIC-2) and cytochrome *b* (*cytb*) genes from genomic DNA, and the *Eimeria tenella* cation-transport ATPase (*Et*Cat ATPase) gene from cDNA were obtained for genotyping using multi-sequence alignments. Finally, sensitivity of the field isolates to three commonly used anticoccidial drugs (diclazuril, decoquinate and maduramycin) were tested to assess the prevalence of drug resistance in *E. tenella* in Hubei Province of China.

**Results:**

Analysis of the ITS1 sequences indicated that all the isolates were *E. tenella*. RAPD analysis and multi-sequence alignments of the SAG, MIC-2, *Et*Cat ATPase and *cytb* showed genetic diversity among these isolates. Finally, drug sensitivity tests demonstrated that all field isolates were sensitive to diclazuril but resistant to decoquinate (except for the isolates from eastern Hubei) and maduramicin.

**Conclusions:**

Population genetic analysis indicated that genetic polymorphisms among field isolates were closely related with their regional distributions. Drug sensitivity testing demonstrated that *E. tenella* isolates in Hubei Province were sensitive to diclazuril, but resistant to maduramycin and decoquinate. The results presented here provide important information for the control and preventions of coccidiosis in the Hubei Province of China.

**Electronic supplementary material:**

The online version of this article (doi:10.1186/s13071-017-2067-y) contains supplementary material, which is available to authorized users.

## Background

Avian coccidiosis, an intestinal parasitic disease caused by *Eimeria* spp., has caused great economic losses to the poultry industry worldwide [1, 2]. When chickens are infected with *Eimeria tenella*, clinical signs include lethargy, feather dishevelment and bloody feces. The main pathological changes include thickening of the intestinal wall and petechial hemorrhages.

*Eimeria tenella* is one of the most pathogenic species of *Eimeria*. Accurate identification is essential for the prevention and control of *E. tenella*. Many studies have focused on genetic diversity of *E. tenella* [3, 4]. As a useful molecular marker, the ITS1 fragment has been widely used for species identification of *Eimeria* [5]. Clark et al. used phylogenetic analysis of ITS sequence data to define species diversity between and within populations for all seven *Eimeria* species of chickens [6]. Schwarz et al. examined the genetic diversity of *Eimeria* species in Arkansas (AR) and North Carolina (NC) by analyzing ITS [7]. Williams et al. developed a RAPD technique based on the amplification of undefined targets by arbitrary primers to detect genetic polymorphisms [8], the technique has been widely used for the analysis of *Eimeria* genetic diversity [9, 10]. Such analysis can help us to estimate the phylogenetic relationship among different *Eimeria* isolates [11].

Currently, the main method to control *Eimeria* infection is anticoccidial drugs [12, 13]. Maduramycin, decoquinate and diclazuril are three chemotherapeutic agents. Maduramycin is thought to kill coccidium by interrupting their normal intracellular ion balance and influence of Na^+^-K^+^-ATPase activity [14]. Decoquinate interferes with the electron transport in the mitochondrial cytochrome system and CytB is an important part of the system [15]. The working mechanism of diclazuril is still unknown. Because of the prolonged use of anticoccidial drugs, resistance to such drugs has been frequently reported [16]. In addition, MIC2 and SAG genes are thought to be involved in host cell adhesion and invasion [17, 18]. Therefore, we aimed to compare the genetic diversity among field isolates using the four selected genes: *Et*Cat ATPase and *cytb* genes which are drug targets; MIC2 and SAG genes which are important for interactions between parasites and host.

In the present study, the genetic diversity and drug sensitivity of *E. tenella* field isolates from Hubei Province of China were analyzed. The results should provide useful information for prevention and control of coccidiosis in this region.

## Methods

### Animals

Coccidia-free, 0-day-old chickens were purchased from Charoen Pokphand Group (Wuhan, China). Chickens were housed in a clean, coccidia-free environment in an isolated brooder room, and fed with commercial broiler feed and water [19].

### Parasite material

Faecal samples were collected from eight different local commercial broiler production farms in Hubei Province between January 2012 and November 2013 (Additional file 1: Table S1). *Eimeria* oocysts obtained from the faecal samples were purified by saturated sodium nitrate flotation and sporulated using standard procedures [20].

Specific pathogens free (SPF) chickens (14-day-old) obtained from Charoen Pokphand Group were orally inoculated with 5 × 10^4^ sporulated oocysts. Seven days post-inoculation, the chickens were sacrificed and necropsies were conducted. The caecal contents were collected for the isolation of *Eimeria* oocysts using the protease digestion method. The protease digestion and sporulation method were as follow: (i) the caeca from *Eimeria* oocysts infected groups were homogenized using a tissue grinder and 2 mg/ml of trypsin was added; (ii) this suspension was incubated in a water bath kettle for 1 h at 39 °C, sieved (180 diameter mesh) and transferred into a 500 ml centrifuge tube; (iii) the suspension was centrifuged at 1000× *g* for 5 min; (iv) the sediment was suspended in 2% w/v aqueous potassium dichromate and transferred to a 50 ml conical tube for oocysts sporulation in an aerobic incubator for 4 days at 28.6 °C. Sporulated oocysts were stored in 2.5% potassium dichromate at 4 °C [21] and were enumerated using the McMaster’s method under the microscope [22].

### DNA and RNA extraction

For DNA extraction, 10^4^ sporulated oocysts from each sample were washed with 1 mM sodium hypochlorite solution for 10 min at 4 °C, then washed three times with deionized water. The oocyst walls were ruptured using a grinding tissue homogenizer for released sporozoites (the volume 200 μl and homogenized), then 100 μl (1 mg/ml) of chicken bile, 100 μl of buffer suspension solution GA, and 20 μl (20 mg/ml) of proteinase K (Genomic DNA Kit, TIANGEN, Beijing, China) were added and the mixture was incubated at 56 °C for 2.5 h. DNA was extracted using a Genomic DNA Kit (TIANGEN), according to the manufacturer’s instructions. DNA concentration was measured using a NanoDrop 2000 nanodrop spectrophotometer (Thermo Fisher Scientific, Waltham, MA, USA). In parallel, total RNA from sporozoites (5 × 10^4^) was extracted using TRIzol reagent, according to the manufacturer’s instructions (Invitrogen, Carlsbad, CA, USA). First strand cDNA was synthesized by a reverse transcription (RT) reaction using the RevertAid First Strand cDNA Synthesis Kit (Thermo Fisher Scientific, Waltham, MA, USA).

### ITS1 amplification and RAPD analysis

Extracted DNA was used as a template to amplify the ITS1 region of *Eimeria* spp., as described by Schnitzler et al. [23, 24]. RAPD random primers were synthesized by Sangon Biotech (Shanghai, China), and the conditions used for RAPD typing were the same as described by Fernandez et al. [10]. The sequences of the random primers used for RAPD amplification are listed in Additional file 1: Table S2. To examine the amplification results, all amplicons were assessed by electrophoresis through 1% agarose gels and then visualized by ethidium bromide staining.

### Polymorphism analysis

Polymorphisms in *cytb*, MIC-2, SAG and *Et*Cat ATPase genes were identified by sequencing the corresponding genes. The *cytb* and SAG genes were PCR amplified from the genomic DNA of isolated isolates using PrimeSTAR® Max DNA Polymerase (TaKaRa, Tokyo, Japan). The primers used are listed in Additional file 1: Table S3 and the following PCR program was used: initial denaturation at 94 °C for 5 min, followed by 35 cycles of 30 s denaturation at 94 °C, 30 s annealing at primer-dependent temperatures, and 60 s extension at 72 °C, followed by a final 7 min extension at 72 °C. The annealing temperatures were 60 °C for the *cytb* gene, 58 °C for MIC-2 and 55 °C for SAG. *Et*Cat ATPase gene sequence is unavailable, the cation-transporting ATPase gene is highly conserved across *Eimeria* species, therefore, the primers used for amplifying *E. tenella* cation-transporting ATPase gene were designed based on the *E. acervulina* clone Dui-10 cation-transporting ATPase gene complete coding region (EU590120.1). The *Et*Cat ATPase gene were amplified from the cDNA of field isolates for sequence alignments using similar PCR reaction conditions. All PCR products were subject to agarose gel electrophoresis and visualized by ethidium bromide staining. PCR products for ITS-1 amplification and polymorphism analysis were send to Sangon Biotech (Shanghai, China) for sequencing using primers in both directions.

### Drug sensitivity tests

Selected field isolates were tested for their sensitivity to three coccidiostat drugs maduramycin, decoquinate and diclazuril. The design of drug-sensitivity tests in Additional file 1: Table S4. In a coccidia-free environment, 170 14-day-old SPF chickens were weighed and randomly divided into 17 groups, each containing ten chickens. All 17 groups were inoculated with *E. tenella* except one, which was not infected and used as a control. Infected chickens were given 5 mg/kg maduramycin, or 1 mg/kg diclazuril, or 30 mg/kg decoquinate, or no treatment as control, in fodder from the time of infection. Chicken faeces were collected from each post-infection groups between 5–7 days single droppings per day to evaluate the relative number of oocysts per gram of feces (OPG). At 21-day of age (7 days post-infection), all the chickens were individually weighed, sacrificed, and necropsied. The weight gain of chickens was recorded. The coccidial lesions present in the chickens were scored as category 0–4 following the methods described by Johnson & Reid [25]. Subsequently the anti-coccidial index (ACI) was calculated to assess drug effectiveness.

### Sequence analysis

Alignment of the ITS1 sequences from *Eimeria* isolates was performed using MAFFT version 7 (http://mafft.cbrc.jp/alignment/server/index.html). Pairwise percentage identity was determined using BioEdit v.7.0 (http://www.mbio.ncsu.edu/bioedit/bioedit.html). RAPD results are analyzed by using SAHN program of the NTSYS-pc software (version 2.02 K,

Applied Biostatistics, Inc, NY, USA). The amino acid sequences of the *Et*Cat ATPase gene from selected parasites were compared by the DNAMAN software (http://www.lynnon.com/dnaman.html). Phylogenetic analyses were conducted using MEGA, version 5.0.

## Results

### Isolation and species identification of *Eimeria tenella* field isolates from local farms in China

**Fig. 1:**
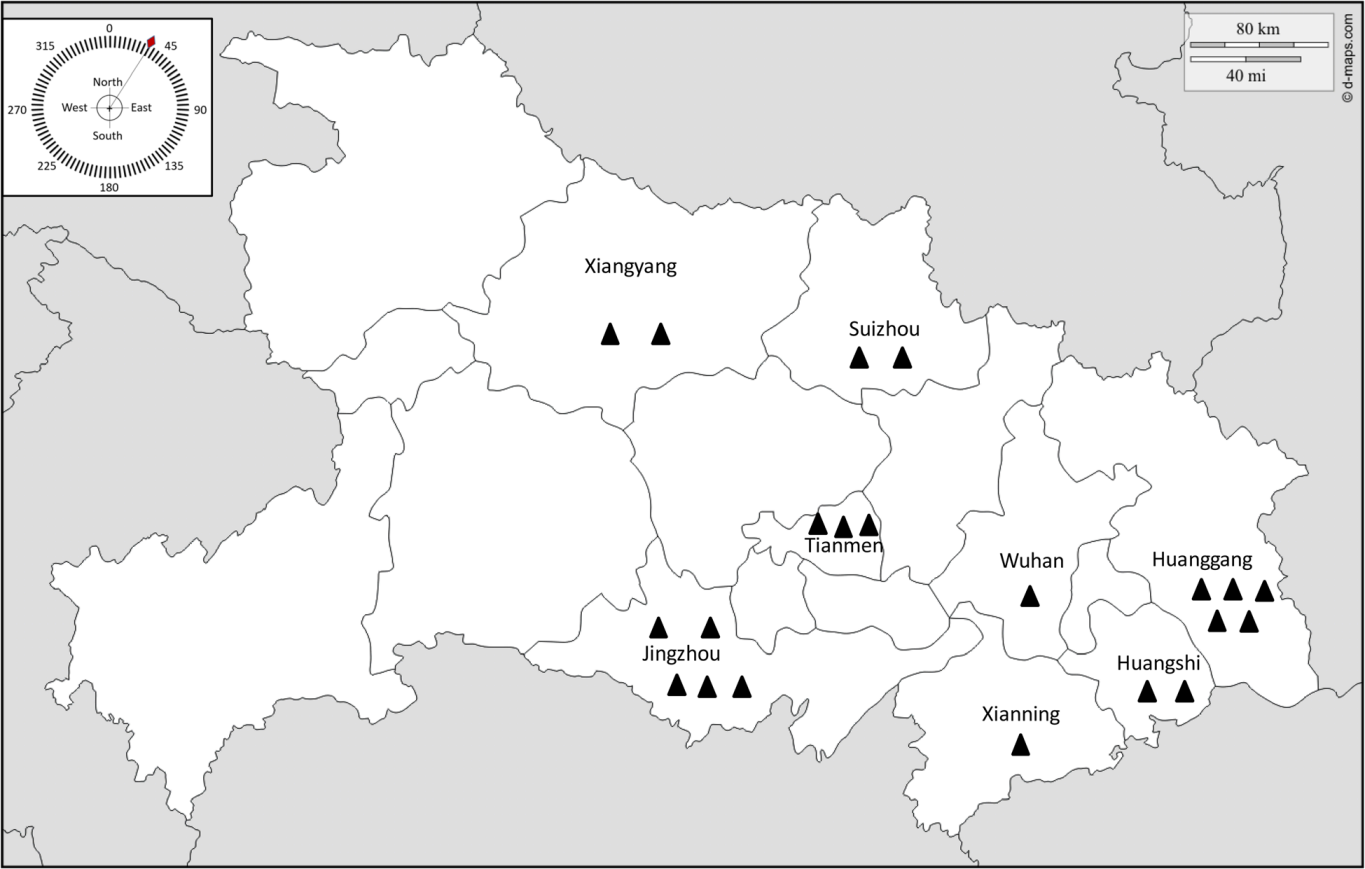
Local commercial broiler farms in Hubei Province from which *Eimeria* isolates were isolated. Faecal samples were collected from different local and different commercial broiler production farms (▲)

**Fig. 2:**
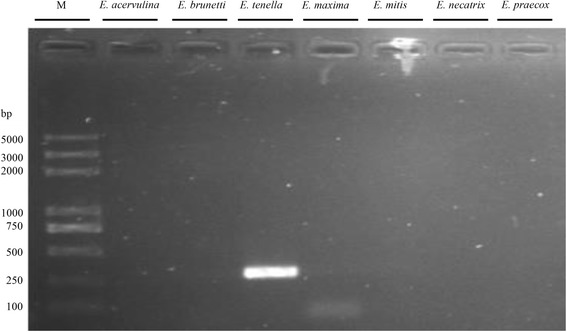
Identification of *Eimeria* species using species-specific primers amplifying the internal transcribed spacer 1 (ITS1) region. Seven pairs of primers specific for *E. acervulina*, *E. brunetti*, *E. mitis*, *E. necatrix*, *E.maxima*, *E.praecox*, and *E.tenella* were used to amplify the genomic DNA of field isolates, and the products were analyzed by agrose gel electrophoresis. One strain is shown as an example. Lane M: Trans2K Plus DNA Marker

**Fig. 3:**
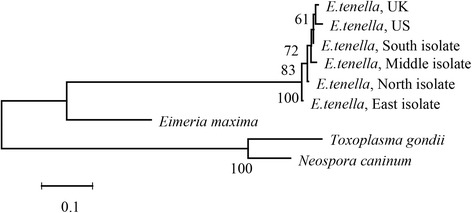
Phylogenetic relationships of *E. tenella* north (Suizhou isolate), *E. tenella* east (Huanggang isolate), *E. tenella* south (Jingzhou isolate) and *E. tenella* middle (Tianmen isolate) with *E. tenella* UK (GenBank: LN609779), *E. tenella* US (GenBank: LN609784), *E. maxima* (GenBank: AF065095), *Neospora caninum* (GenBank: AF029702) and *Toxoplasma gondii* (GenBank: L49390); based on ITS1 sequences by neighbor-joining analysis. Nodal support values are indicated (%). The scale-bar indicates sequence substitution per site

Twenty-one *Eimeria* field samples were isolated from different local commercial broiler production farms in Hubei Province (Fig. 1). To confirm the species identity of these *Eimeria* samples, seven pairs of species-specific primers (for *E. acervulina*, *E. brunetti*, *E. mitis*, *E. necatrix*, *E. maxima*, *E. praecox* and *E. tenella*, respectively) amplifying ITS1 region were used. The identification results of *Eimeria* species using species specific primers amplifying the internal transcribed spacer 1 (ITS1) region in Additional file 1: Table S5. The results indicate that all the field isolates were *E. tenella* since they produced a specific 278 bp band using the *E. tenella*-specific primer (one example is given in Fig. 2). This fragment obtained from each strain was subject to DNA sequencing and the results showed that they were 97–100% identical to the ITS1 sequence of *E. tenella* (GenBank No. AF026388), further confirming that these isolates are *E. tenella* (GenBank No. KY117132–KY117152). Pairwise comparison of ITS1 sequences from these isolates revealed high sequence identities ranging from 92.5–100% (Table 1), suggesting homology among these isolates. Phylogenetic relationship based on the ITS1 of *E. tenella* north (Suizhou isolate), *E. tenella* east (Huanggang isolate), *E. tenella* south (Jingzhou isolate), *E. tenella* middle (Tianmen isolate), *E. tenella* UK (GenBank: LN609779), *E. tenella* US (GenBank: LN609784), *E. maxima* (GenBank: AF065095), *Neospora caninum* (GenBank: AF029702) and *Toxoplasma gondii* (GenBank: L49390) was accomplished by MEGA v6.0 using neighbor-joining method with default setting [26], and nodal support values were indicated (%). The phylogenetic tree demonstrated the four *E. tenella* field isolates had close relationship with *E. tenella* UK (Fig. 3).

**Table 1:** Pairwise comparison between the ITS-1 sequences of different *Eimeria tenella* isolates

Seq->	TM1	TM2	TM3	SZ1	SZ2	HS	JZ1	JZ2	JX	XN	XS1	XS2	XS3	XS4	XS5	SS1	SS2	SS3	XY1	XY2	DY
TM1																					
TM2	94.8																				
TM3	95.6	94.8																			
SZ1	96.8	95.6	97.6																		
SZ2	96.0	95.6	97.2	96.8																	
HS	94.8	96.4	94.8	95.6	95.2																
JZ1	97.6	95.2	96.8	98.0	97.2	96.4															
JZ2	94.9	93.7	94.5	94.9	94.9	94.9	95.7														
JX	96.0	94.4	95.2	96.0	96.8	95.6	97.6	95.3													
XN	95.6	95.2	96.0	96.0	96.0	94.5	96.8	94.9	95.7												
XS1	94.4	96.4	96.0	96.4	95.6	96.0	95.6	94.5	94.4	94.9											
XS2	97.2	95.6	97.2	98.4	97.6	96.4	99.6	95.7	97.6	97.2	96.0										
XS3	97.2	95.2	97.2	97.2	97.2	95.6	98.4	95.7	96.4	96.8	95.6	98.8									
XS4	96.8	96.0	96.8	97.2	98.4	96.0	98.4	96.4	97.2	97.2	96.4	98.8	98.0								
XS5	96.8	94.4	96.0	96.8	96.4	95.2	98.8	95.7	96.4	96.0	96.0	98.4	97.6	97.6							
SS1	92.5	94.0	94.8	93.6	93.6	94.8	94	92.6	93.7	93.7	96.0	94.4	94.0	94.0	94.1						
SS2	94.1	95.2	95.6	95.2	95.2	95.6	95.2	94.9	94.8	94.9	96.8	95.6	95.2	96.0	94.5	97.2					
SS3	96.8	95.6	98.3	97.6	97.2	94.8	97.2	94.5	96.4	96.0	96.0	97.6	96.8	96.8	96.0	94.4	95.6				
XY1	96.8	95.6	97.2	98.0	96.4	95.6	98	94.5	96.0	96.0	95.6	98.4	97.2	97.2	96.8	94.0	94.8	98.0			
XY2	95.2	96.4	96.4	96.0	98.4	96.0	96.4	94.1	96.0	95.2	96.4	96.8	96.0	97.2	96.0	95.2	96.4	96.8	95.6		
DY	93.3	94.0	95.2	94.0	94.4	95.2	94.4	94.1	94.0	93.7	96.0	94.8	94.4	95.2	94.5	97.6	98.0	94.4	93.6	96.0

### RAPD analysis

**Fig. 4:**
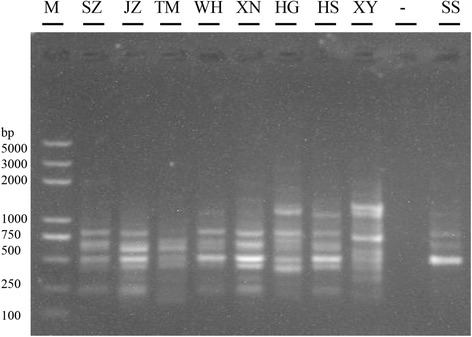
Random amplified polymorphic fragments obtained with decamer primers S2133 using DNA samples of different field isolates. *Abbreviations*: SZ, Suizhou; JZ, Jingzhou; TM, Tianmen; WH, Wuhan; XN, Xianning; XS, Xishui; HS, Huangshi; XY, Xiangyang; SS, Shashi; “-”, Negative control; M: Trans2K Plus DNA Marker

**Fig. 5:**
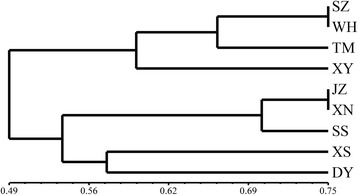
Clustering of *E. tenella* field isolates following RAPD analysis. *Abbreviations*: SZ, Suizhou; JZ, Jingzhou; TM, Tianmen; WH, Wuhan; XN, Xianning; XS, Xishui; HS, Huangshi; XY, Xiangyang; SS, Shashi

To optimize the primers for RAPD analysis, 70 decamer were screened using *E. tenella* DNA samples. After the initial screening, seven primers were found to discriminate the *E. tenella* species. They resulted in two to eight amplicons ranging from 250–1,500 bp using the field isolates as templates. The amplification patterns of the field isolates generated by the S2133 primer are shown in Fig. 4. NTSYS-pc software was used to convert the amplification results into data to assess the phylogenetic relationships among the *E. tenella* field isolates (Fig. 5). Briefly, the unweighted pair-group method with arithmetic averages (UPGMA) dendrogram of the RAPD data was calculated and analysised using SAHN program of the NTSYS-pc software. The RAPD data were generated from the DNA fingerprints of the eight *E. tenella* isolates using seven random primers, and the results indicated eight *E. tenella* isolates were separated into two branch clusters mainly based on geographical distribution, the first cluster included SZ, XY, TM and WH, and the second cluster included JZ, SS, XS and HG. The phylogenetic branch lengths ranged from 0.49 to 0.75, and the clusters may be caused by cross-regional transportation of animals.

### Sequence diversity of the cytochrome B gene

Amplicons of the *E. tenella cytb* gene (1,268 bp) derived from the field isolates were sequenced and aligned with that of the *E. tenella* Houghton strain (GenBank: HQ173891.1). The result revealed that the *cytb* gene sequences from our field isolates (GenBank: KY117213–KY117232) have 99.9% similarity with the *cytb* gene from the *E. tenella* Houghton strain. Polymorphisms were observed at three positions (position 22, C-A; position 49, A-G; position 838, T-C); these are listed in Table 2. These nucleotide changes do result in amino acid alterations in the CytB protein.

**Table 2:** Mutations in the CytB gene of *Eimeria tenella* field isolates

Strains	Mutations
DY	C-A(22^th^) A-G(49^th^) T-C(838^th^)
XS1	C-A (22^th^)
XS2	C-A (22^th^)
XS3	0
XS4	0
HS	0
JZ1	C-A/C(22^th^) A-G/A(49^th^) T-C(838^th^)
JZ2	C-A/C(22^th^) A-G/A(49^th^) T-C(838^th^)
SS1	C-A/C(22^th^) A-G/A(49^th^) T-C(838^th^)
SS2	C-A/C(22^th^) A-G/A(49^th^) T-C(838^th^)
SS3	C-A(22^th^) A-G(49^th^) T-C(838^th^)
SZ1	C-A/C(22^th^) A-G/A(49^th^) T-C(838^th^)
SZ2	C-A/C(22^th^) A-G/A(49^th^) T-C(838^th^)
TM1	C-A(22^th^) A-G(49^th^) T-C(838^th^)
TM2	C-A(22^th^) A-G(49^th^) T-C(838^th^)
TM3	C-A(22^th^) A-G(49^th^) T-C(838^th^)`
WH	C-A(22^th^) A-G(49^th^) T-C/T(838^th^)
XN	C-A(22^th^) A-G(49^th^) T-C(838^th^)
XY1	C-A(22^th^) A-G(49^th^) T-C(838^th^)
XY2	C-A(22^th^) A-G(49^th^) T-C(838^th^)

### Sequence diversity of the MIC-2 and SAG genes

Amplification of the MIC-2 gene from the *E. tenella* field isolates produced a specific 1,647-bp band. This fragment from each isolate was sequenced and aligned with the *E. tenella* MIC-2 gene (GenBank: AF111702.1). The results indicated that there is no polymorphism between field isolates (GenBank: KY117173 to KY117192) and the reference strain. Amplification of SAG gene yielded a single 1,101-bp band in all field isolates. Sequence alignment with the *E. tenella* surface antigen gene (GenBank: M21088.1) indicated that all of the field isolates (GenBank: KY117193–KY117212) contained two nucleotide substitutions at positions 209 (A-G) and 901 (C-G), but these nucleotide substitutions did not cause amino acid changes.

### *Et*Cat ATPase gene

**Fig. 6:**
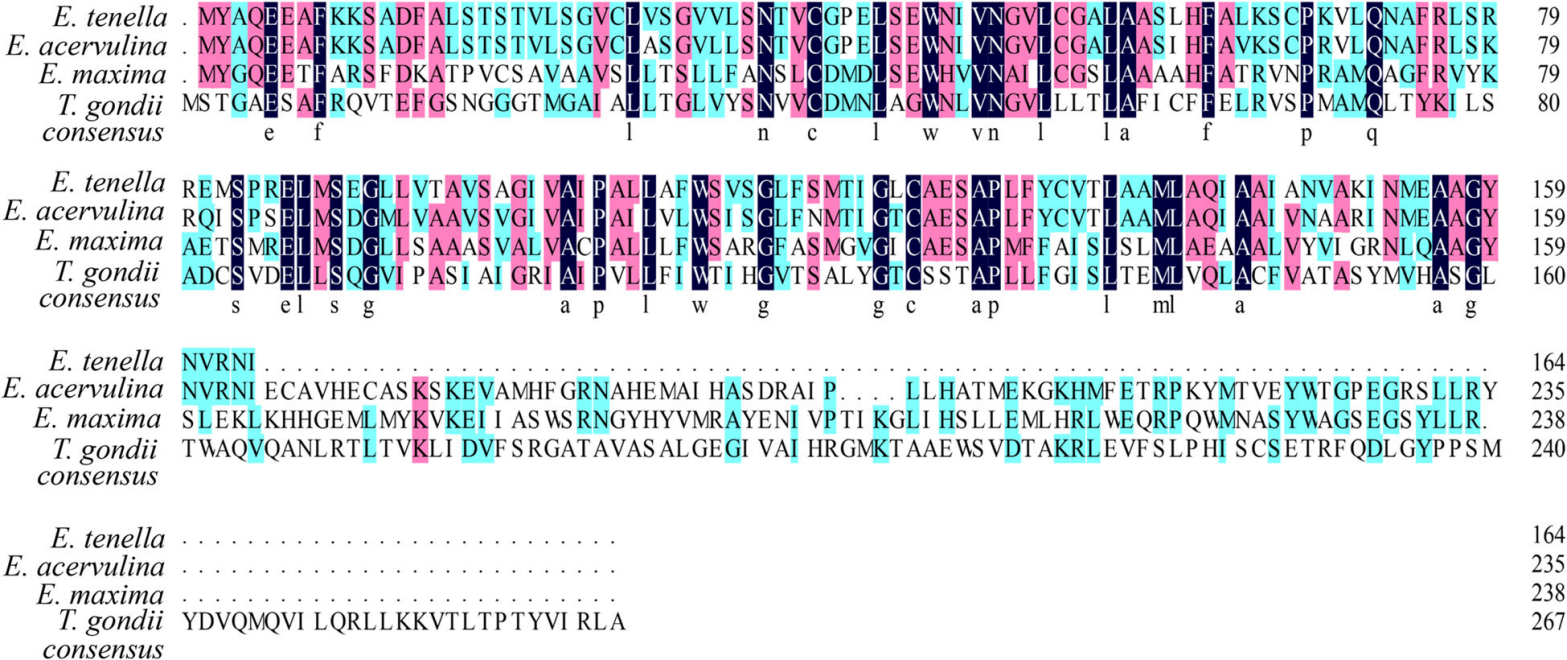
Sequence alignment of *E. tenella* Cation-transport ATPase with its homologs from selected parasites. *Black*: 100% amino acid identity; *red*: 75% amino acid identity; *blue*: 50% amino acid identity

PCR amplification of the *Et*Cat ATPase gene from the field isolates produced one single band of 494 bp on agarose gels. Sequencing analysis indicate that the sequences of *Et*.Cat ATPase gene from all field isolates (GenBank: KY126385 to KY126404) are also identical to the *E. tenella* mitochondrial hypothetical protein (GenBank: KF670727.1). The amino acid sequence of *Et*.Cat ATPase shared 87% similarity with that of the *E. acervulina* cation transporter ATPase gene (GenBank: EU590120.1), 80% similarity with *E. maxima* Cation-transporting ATPase (GenBank: XP_013337038.1), and 34% similarity with the cation-transporting ATPase gene (GenBank: EPR61390.1) of *T. gondii* (Fig. 6).

### Drug susceptibility of *E. tenella* isolates from Hubei Province

To test the status of drug resistance in the field, four isolates representing different geographical origins were selected and their sensitivity to commercially available drugs were tested. Diclazuril had an ACI higher than 180 in the field isolates from northern, eastern and middle regions of Hubei Province. The ACI of Diclazuril on the strain from southern part of Hubei is 168.8. These results suggest that all field isolates from these regions are sensitive to Diclazuril. The data for decoquinate drug tests showed that *E. tenella* field isolates from northern, southern and middle Hubei had ACI below 160, suggesting decoquinate tolerance. While for the strain from eastern Hubei, decoquinate had an ACI value of 175.8, indicating that this isolates is still sensitive to decoquinate. For maduramicin, and all tested isolates had ACI values below 160, which indicate that they were resistant to this drug (Table 3).

**Table 3:** The drug susceptibility of *E. tenella* field isolates

Groups	Field isolates	Weight gain (g)	Weight gain of NNC^g^ (%)	Survival rate (%)	Lesion score	OPG (10^5^)	Anticoccidial index
1^a^	North isolate^d^	76	100	100	1.2	0.813	183
2^a^	East isolate^e^	77	101.3	100	1.0	0.658	186.3
3^a^	South isolate^f^	69	90.8	90	1.2	1.44	168.8
4^a^	Middle isolate^g^	77	101.3	100	1.1	0.792	185.3
5^b^	North isolate^d^	54	71.1	100	2.6	1.06	140.1
6^b^	East isolate^e^	66	86.8	100	0.6	0.598	175.8
7^b^	South isolate^f^	47	61.8	100	1.6	1.27	140.8
8^b^	Middle isolate^g^	62	81.6	70	3.2	1.3	129.6
9^c^	North isolate^d^	53	69.7	100	2.8	0.938	136.7
10^c^	East isolate^e^	55	73.3	100	2.1	0.957	147.3
11^c^	South isolate^f^	51	67.1	90	2.4	1.16	128.1
12^c^	Middle isolate^g^	50	65.8	90	3.1	1.25	119.8
13	North control	39	51.3	90	3.5	6.25	108.8
14	East control	63	82.9	80	3.4	5.98	123.5
15	South control	46	60.5	90	3.4	5.52	114.1
16	Middle control	44	57.9	80	3.3	5.66	98.6
17^h^	NNC control	76	100	100	0	0	200

^a^Diclazuril treatment groups

^b^Decoquinate treatment groups

^c^Maduramycin treatment groups

^d^Suizhou isolate

^e^Huanggang isolate

^f^Jingzhou isolate

^g^Tianmen isolate

^d-g^ isolates control: infected dose per chicken for 5 × 10^4^ sporulated oocysts, non-treatment control (groups, 13–16)

^h^NNC control: non-infected, non-treatment control (group,17)

## Discussion

Chicken coccidiosis caused by *Eimeria* species leads to severe economic losses to the poultry industry worldwide [27, 28]. It is listed as one of the top five most devastating diseases in poultry. The morbidity of coccidiosis is estimated to be 50–70% and the disease is a major threat to 15–50 day-old chickens [29–31].

In this study, we purified *Eimeria* oocysts from chicken faecal samples collected from eight regions (Suizhou, Xiangyang, Huanggang, Huangshi, Xianning, Wuhan, Jingzhou, Tianmen) in Hubei Province of China. Subsequently all the isolates were identified to be *E. tenella* using species-specific PCR amplification of the ITS1 rDNA region.

The PCR-based RAPD technique, which was originally developed in the 1990s, can be used to genotype organisms and identify unknown sequence polymorphisms among genetically diverse isolates [32]. Here, we used random primers designed by Sangon Biotech for RAPD analysis of the *E. tenella* field isolates collected from different regions of Hubei, China. The result indicated that there are genetic differences among the *E. tenella* field isolates in this region and the differences may correlate with geographical origin of the isolates.

Many studies have investigated the development of resistance mechanisms against anti-coccidian drugs in *Eimeria* parasites and proposed various hypotheses [33]. However, the most popular opinion for the development of drug resistance is the endogenous mutations occurring in coccidian parasite [34]. Because of continuous and prolonged use of anticoccidial drugs, mutations have been observed among coccidian population during this continuous selection process. These mutations maybe involved in the development of drug resistance [35].

In other apicomplexan species, genetic mutations are known to be associated with drug resistance. The correlation between the acquisition of mutations and drug resistance has been verified, for example, a point mutation at position 268 of the *cytb* gene was reported to induce atovaquone resistance in *Plasmodium falciparum* [36]. In the present study, genetic mutations also existed in resistant field isolates, whether the mutations are related with drug resistance of field isolates or not need further verification.

In addition to RAPD, we also examined the genetic diversity of field isolates by checking the sequence polymorphisms in MIC-2, SAG, *Et*Cat ATPase and *cytb* genes. Sequence analysis indicated that the MIC-2, SAG, *Et*Cat ATPase genes are highly conserved among *E. tenella* isolates. For the *cytb* gene, the alignments showed that some isolates contain polymorphisms leading to missense mutations. These changes occurred at positions eight, 17 and 280 of the CytB protein.

## Conclusions

In this study, we isolated and analyzed *E. tenella* field isolates from eight regions of Hubei Province, P. R. China. RAPD analysis and multiple sequence alignments of SAG, MIC-2, *cytb* and *Et*Cat ATPase revealed genetic diversity among field isolates. The drug sensitivity tests indicated that maduramycin and decoquinate resistance are widely present in Hubei, but diclazuril is still effective towards *E. tenella* isolates in this region. These results are important for the selection of strategies to control chicken coccidiosis in this region.

## Additional file

Additional file 1: Table S1. Local commercial broiler farms in Hubei province from which *Eimeria* isolates were isolated. **Table S2.** Primers used in RAPD analysis by Sangon Biotech, Shanghai. **Table S3.** Primers used for PCR amplification of indicated genes. **Table S4.** The design of drug-sensitivity tests. **Table S5.** Identification results of *Eimeria* species using species specific primers amplifying the internal transcribed spacer 1 (ITS1) region. (DOC 126 kb)

## Abbreviations

RAPD: Random amplified polymorphic DNA
ITS: Internal transcribed spacer 1
SAG: Surface antigen
MIC-2: Micronemal protein 2
*cytb*: Cytochrome *b*
*Et*Cat ATPase: *Eimeria tenella* cation-transport ATPase
RT: Reverse transcription
OPG: Oocysts per gram of faeces
SPF: Specific pathogens free
ACI: Anti-coccidial index
