# Supplementary material for: Genetic diversity and drug sensitivity studies on Eimeria tenella field isolates from Hubei Province of China
Source: Parasit Vectors. 2017 Mar 9;10:137. doi: 10.1186/s13071-017-2067-y (PMC5343410; doi:10.1186/s13071-017-2067-y)
Supplement: Additional file 1: Table S1. — Local commercial broiler farms in Hubei province from which Eimeria isolates were isolated. Table S2. Primers used in RAPD analysis by Sangon Biotech, Shanghai. Table S3. Primers used for PCR amplification of indicated genes. Table S4. The design of drug-sensitivity tests. Table S5. Identification results of Eimeria species using species specific primers amplifying the internal transcribed spacer 1 (ITS1) region. (DOC 126 kb) [file 13071_2017_2067_MOESM1_ESM.doc]

**Additional file 1**

**Table S1.** Local commercial broiler farms in Hubei province from which *Eimeria* isolates were isolated

| *Eimeria* isolates | source | regional distribution | collection time |
| --- | --- | --- | --- |
| DY | Huang shi | East | 2012.03 |
| XS1 | Huang gang | East | 2013.03 |
| XS 2 | Huang gang | East | 2013.03 |
| XS 3 | Huang gang | East | 2013.03 |
| XS 4 | Huang gang | East | 2013.03 |
| XS 5 | Huang gang | East | 2013.03 |
| JZ1 | Jing zhou | South | 2013.07 |
| JZ2 | Jing zhou | South | 2013.07 |
| SS1 | Sha shi | South | 2013.07 |
| SS2 | Sha shi | South | 2013.07 |
| SS3 | Sha shi | South | 2013.07 |
| XN | Xian ning | South | 2012.11 |
| SZ1 | Sui zhou | North | 2013.09 |
| SZ2 | Sui zhou | North | 2013.09 |
| HS | huangshi | East | 2013.03 |
| TM1 | Tian men | Middle | 2012.1 |
| TM2 | Tian men | Middle | 2012.1 |
| TM3 | Tian men | Middle | 2012.1 |
| JX | Wu han | Middle | 2012.09 |
| XY1 | Xiang yang | North | 2013.09 |
| XY2 | Xiang yang | North | 2013.09 |

**Table S2.** The primers used in RAPD analysis by Sangon Biotech, Shanghai

|  | Number | Primer name | Primer sequence 5'→3' | Number | Primer name | Primer sequence 5'→3' |
| --- | --- | --- | --- | --- | --- | --- |
|  | 1 | S6 | TGCTCTGCCC | 36 | S509 | TGAGCACGAG |
|  | 2 | S7 | GGTGACGCAG | 37 | S1001 | TCCGCAACCA |
|  | 3 | S8 | GTCCACACGG | 38 | S1034 | TGGTGCACTC |
|  | 4 | S9 | TGGGGGACTC | 39 | S1035 | GACACAGCCC |
|  | 5 | S22 | TGCCGAGCTG | 40 | S1036 | AAGGCACGAG |
|  | 6 | S23 | AGTCAGCCAC | 41 | S1081 | TGTGACGAGG |
|  | 7 | S49 | CTCTGGAGAC | 42 | S1082 | ACCCTGTGGG |
|  | 8 | S50 | GGTCTACACC | 43 | S1156 | CACAACGGGA |
|  | 9 | S51 | AGCGCCATTG | 44 | S1157 | CCCATGTGTG |
|  | 10 | S52 | CACCGTATCC | 45 | S1158 | GGGAGCGCTT |
|  | 11 | S74 | TGCGTGCTTG | 46 | S1222 | GTCCTCGTGT |
|  | 12 | S75 | GACGGATCAG | 47 | S1223 | ACGGTTCCAC |
|  | 13 | S82 | GGCACTGAGG | 48 | S1224 | GTCTTGGGCA |
|  | 14 | S83 | GAGCCCTCCA | 49 | S1225 | GTCACCTGCT |
|  | 15 | S108 | GAAACACCCC | 50 | S1317 | TGCTGCTGCC |
|  | 16 | S109 | TGTAGCTGGG | 51 | S1318 | GGCGCAACTG |
|  | 17 | S135 | CCAGTACTCC | 52 | S1319 | GGACACAGAG |
|  | 18 | S136 | GGAGTACTGG | 53 | S1320 | TGTCCTAGCC |
|  | 19 | S202 | GGAGAGACTC | 54 | S1415 | CCTCCTTCTC |
|  | 20 | S203 | TCCACTCCTG | 55 | S1418 | CTGGCGTGTC |
|  | 21 | S215 | GGATGCCACT | 56 | S1488 | CTCTGCCTGA |
|  | 22 | S249 | CCACATCGGT | 57 | S1517 | AGCGGAAGTG |
|  | 23 | S250 | ACCTCGGCAC | 58 | S1518 | GTGGGCATAC |
|  | 24 | S277 | GTCCTGGGTT | 59 | S2046 | GAAGGCGAGA |
|  | 25 | S324 | AGGCTGTGCT | 60 | S2049 | GTCATGCGAC |
|  | 26 | S325 | TCCCATGCTG | 61 | S2076 | GAACTCCCAG |
|  | 27 | S326 | GTGCCGTTCA | 62 | S2078 | CGCACACTC |
|  | 28 | S338 | AGGGTCTGTG | 63 | S2110 | GTGACCAGAG |
|  | 29 | S349 | TGAGCCTCAC | 64 | S2133 | GGTTGGGCCA |
|  | 30 | S371 | AATGCCCCAG | 65 | S2137 | TCCGGGACTC |
|  | 31 | S372 | TGGCCCTCAC | 66 | S2145 | GTAGGTCGCA |
|  | 32 | S437 | CATTGGGGAG | 67 | S2146 | TCGTGGCACA |
|  | 33 | S438 | GGTGAGGTCA | 68 | S2157 | CTCTTACGGG |
|  | 34 | S477 | TGACCCGCCT | 69 | S2158 | GACGCTTGTC |
|  | 35 | S481 | GGGACGATGG | 70 | S2160 | CACCGACATC |

**Table S3. Primers used for PCR amplification of indicated genes**

| Gene | Primer reference | Primer sequence 5'→3' | Expected product size (bp) | Genebank Accession No. |
| --- | --- | --- | --- | --- |
| CytB | CytBF | ATGTCTCAAGTGAGATCTCA | 1080 bp | HQ173891.1 |
|  | CytBR | TTACAGTTTGAATAAACTAA |  |  |
| MIC2 | MIC2F | ATGGCTCGAGCGTTGTCGCTGG | 1647 bp | AF111702.1 |
|  | MIC2R | TCAGGATGACTGTTGAGTGTCACTC |  |  |
| SAG | SAGF | ATGGCTCGTCTTTCTTTTGTTTCTC | 1101 bp | M21088.1 |
|  | SAGR | CTAAAAGAGAGCGAAAGCGGAGAT |  |  |
| *Et*Cat  ATPase | ATPaseF | ATGTACGCCCAAGAAGAAGCC | 492 bp | EU590120.1 |
|  | ATPaseR | TCAATGTTGCGGACATTGTATCCAG |  |  |

**Table S4.** The design of drug-sensitivity tests

| Groups | Field isolates | Number of animals | Anticoccidials |
| --- | --- | --- | --- |
| 1 | North isolate^a^ | 10 | Diclazuril |
| 2 | North isolate | 10 | Decoquinate |
| 3 | North isolate | 10 | Maduramycin |
| 4 | North isolate | 10 | No treatment |
| 5 | East isolate^b^ | 10 | Diclazuril |
| 6 | East isolate | 10 | Decoquinate |
| 7 | East isolate | 10 | Maduramycin |
| 8 | East isolate | 10 | No treatment |
| 9 | South isolate^c^ | 10 | Diclazuril |
| 10 | South isolate | 10 | Decoquinate |
| 11 | South isolate | 10 | Maduramycin |
| 12 | South isolate | 10 | No treatment |
| 13 | Middle isolate^d^ | 10 | Diclazuril |
| 14 | Middle isolate | 10 | Decoquinate |
| 15 | Middle isolate | 10 | Maduramycin |
| 16 | Middle isolate | 10 | No treatment |
| 17 | NNC control^e^ | 10 | No treatment |

^a^Suizhou isolate, ^b^Huanggang isolate, ^c^Jingzhou isolate, ^d^Tianmen isolate.

^e^NNC= non-infected,non-treatment control(group,17).

Infected chickens were given 5 mg/kg maduramycin, or 1 mg/kg diclazuril, or 30 mg/kg decoquinate.

**Table S5.** The identification results of *Eimeria* species using species specific primers amplifying the internal transcribed spacer 1 (ITS1) region

| *Eimeria* isolates | Source | *E. acervulina* | *E. brunetti* | *E. maxima* | *E. mitis* | *E. necatrix* | *E. praecox* | *E. tenella* |
| --- | --- | --- | --- | --- | --- | --- | --- | --- |
| DY | Huang shi | *-* | *-* | *-* | *-* | *-* | *-* | *+* |
| XS1 | Huang gang | *-* | *-* | *-* | *-* | *-* | *-* | *+* |
| XS 2 | Huang gang | *-* | *-* | *-* | *-* | *-* | *-* | *+* |
| XS 3 | Huang gang | *-* | *-* | *-* | *-* | *-* | *-* | *+* |
| XS 4 | Huang gang | *-* | *-* | *-* | *-* | *-* | *-* | *+* |
| XS 5 | Huang gang | *-* | *-* | *-* | *-* | *-* | *-* | *+* |
| JZ1 | Jing zhou | *-* | *-* | *-* | *-* | *-* | *-* | *+* |
| JZ2 | Jing zhou | *-* | *-* | *-* | *-* | *-* | *-* | *+* |
| SS1 | Sha shi | *-* | *-* | *-* | *-* | *-* | *-* | *+* |
| SS2 | Sha shi | *-* | *-* | *-* | *-* | *-* | *-* | *+* |
| SS3 | Sha shi | *-* | *-* | *-* | *-* | *-* | *-* | *+* |
| XN | Xian ning | *-* | *-* | *-* | *-* | *-* | *-* | *+* |
| SZ1 | Sui zhou | *-* | *-* | *-* | *-* | *-* | *-* | *+* |
| SZ2 | Sui zhou | *-* | *-* | *-* | *-* | *-* | *-* | *+* |
| HS | huangshi | *-* | *-* | *-* | *-* | *-* | *-* | *+* |
| TM1 | Tian men | *-* | *-* | *-* | *-* | *-* | *-* | *+* |
| TM2 | Tian men | *-* | *-* | *-* | *-* | *-* | *-* | *+* |
| TM3 | Tian men | *-* | *-* | *-* | *-* | *-* | *-* | *+* |
| JX | Wu han | *-* | *-* | *-* | *-* | *-* | *-* | *+* |
| XY1 | Xiang yang | *-* | *-* | *-* | *-* | *-* | *-* | *+* |
| XY2 | Xiang yang | *-* | *-* | *-* | *-* | *-* | *-* | *+* |

“-” indicates negative PCR amplification results using corresponding species specific primers, whereas “+” represents positive PCR amplification.
